# Effects of iodine-125 seeds on the methylation of SFRP_2_ and P16 in colorectal cancer

**DOI:** 10.3892/etm.2013.1298

**Published:** 2013-09-13

**Authors:** XIAOGANG LI, PING LU, BO LI, WANGFU ZHANG, KAIYUAN LUO

**Affiliations:** 1 Departments of General Surgery, The Fourth Affiliated Hospital of Kunming Medical University, Kunming, Yunnan 650021, P.R. China; 2 Vascular Surgery, The Fourth Affiliated Hospital of Kunming Medical University, Kunming, Yunnan 650021, P.R. China

**Keywords:** iodine-125 seeds, colorectal cancer, SFRP_2_, P16, methylation

## Abstract

The current study investigated the effects of iodine-125 seeds on the gene methylation of SFRP_2_ and P16 in colorectal cancer. Mouse models of human colorectal cancer were randomly divided into an experimental group (n=25) and a control group (n=25). The control group was implanted with blank seeds (0 MBq) and the experimental group with iodine-125 seeds (14.8 MBq). At 20 days after implantation, the animals were sacrificed. The methylation levels of SFRP_2_ and P16 were detected using methylation-specific polymerase chain reactions (MSPs). Following iodine-125 seed irradiation, the level of SFRP_2_ methylation decreased. The methylation index of the experimental group (0.67±0.05) was significantly lower than that of the control group (0.84±0.07; P<0.05). In the experimental group, 10 samples (40%) displayed methylation in the P16 promoter region compared with 14 (56%) in the control group, which was a significant difference (P<0.05). Iodine-125 seeds induce the downregulation of methylated tumor suppressor gene promoters, thereby inhibiting the proliferation and growth of tumor cells.

## Introduction

As the social economy develops and lifestyles and food habits change, the incidence rate of colorectal cancer is increasing in China, with an annual increase that has reached as high as 4% since the 1970s. In the US, colorectal cancer has become the third most common cause of cancer-associated mortalities (1). Patients presenting with this disease have a notably shortened life span, as well as a poor quality of life. Radical surgery fails to achieve satisfactory results, with a recurrence rate >33% (2). This rate may reach as high as 90% following radical surgery (3). Even radical surgery combined with intraoperative chemotherapy is only able to achieve a local control rate of 50% (2). For patients with intrapelvic recurrent colorectal cancer, traditional surgery achieves a 3-year survival rate of only 8% (no 5-year survival rate) and a median time of remission from bleeding and pain of between 5 and 6 months (4). Although total pelvic exenteration may achieve a superior curative effect, the 3-year survival rate is no more than 32% (5). The prognosis following palliative surgery is much worse. Colorectal cancer has a high recurrence rate following surgery and is difficult to re-treat. Neither chemotherapy alone nor additional surgery is able to achieve a satisfactory curative effect. Therefore, radiotherapy has attracted an increasing amount of attention.

Radioactive particles have a persistent lethal effect on the uncontrolled proliferation of tumor cells with a definite curative effect on tumors. Radioactive particles may achieve a 3-year survival rate of up to 87.2% (6). Iodine-125 seeds have a long half-life and low energy with excellent stability, which has led to their extensive application in clinical practice. Jarusevicius *et al* (7) first applied permanent implantation with 229 iodine-125 seeds for a malignant neurilemmoma following chemoradiotherapy failures and obtained successful results. Since then, permanent iodine-125 seed implantation has been used as the preferred method for the treatment of prostate cancer in the US, Canada, South Africa and other countries. Iodine-125 seed implantation has also been gradually applied in the treatment of other malignant carcinomas, including breast carcinoma, brain neoplasm, lung carcinoma and hepatocarcinoma (6–8). The authors of the present study have used this technique for >20 types of malignant carcinomas since developing and popularizing it in China for the first time in 1999 (9,10). Brachytherapy with iodine-125 seed implantation has since been reported to have favorable curative effects on tumors, and this technique has become an important method for the treatment of colorectal cancer (11,12). Studies have demonstrated that the development of colorectal cancer is accompanied by the CpG island hypermethylation of numerous tumor suppressor gene promoters and that promoter hypermethylation leads to the downregulation or silencing of tumor suppressor gene expression (13,14). However, the effect of iodine-125 seeds on gene methylation has not been reported.

In the current study, we implanted iodine-125 seeds into mice with colorectal cancer to detect its effects on the methylation of the SFRP_2_ and P16 tumor suppressor genes.

## Materials and methods

### 

#### Animals

Fifty male Balb/c-nu/nu mice aged between 6 and 8 weeks and weighing between 18 and 20 g were purchased from The Beijing Cancer Institute (Beijing, China). The animals had been subcutaneously inoculated with HCT-8 human colorectal cancer cells at the right armpit under specific pathogen free (SPF) conditions and the diameters of the tumors were ~0.5±0.3 cm. After purchase, the mice were fed under SPF conditions.

This study was carried out in strict accordance with the recommendations in the Guide for the Care and Use of Laboratory Animals of the National Institutes of Health (2007). The animal use protocol was reviewed and approved by the Institutional Animal Care and Use Committee of Kunming Medical University (Kunming, China).

### Primer design

#### SFRP_2_ primers

SFRP_2_ primers were designed at http//www.urogene.org/methprimer (accessed November 3, 2011 (15). The following sequence was synthesized: 5′-GTT TTT TTT ATT TTT TAG ATT TGT ATA AAA AAG GTT AAG AAA ATT TTG GTT GTG TTT TAG TAA CGG TTT ATT TTG TTT TTT CGG GTC GGA GTT TTT CGG AGT TGC GCG CGG GTT TGT AGC GTT TCG TTC GCG TTG TTT TTT CGG TGT TTC GTT TTT TCG CGT TTT AGT CGT CGG TTG TTA GTT TTT CGG GGT TTC GAG TCG TAT TTA GCG AAG AGA GCG GGT TCG GGA TAA GTT CGA ATT TCG GTC GTT TCG TTT TTT TTC GGT TTC GTT TTT TTT GTT TTT TCG GGG TCG CGC GTT TAC GAT GTT GTA GGG TTT TGG TTC GTT GTT GTT GTT TTT TTT CGT TTC GTA TTG TTG TTT GGG TTC GGC GCG CGG GTT TTT TTT TTT TGG TTA GTT CGA TTT TTT TTA TAA GCG TAG TAA TTG TAA GTT TAT TTT TGT TAA TTT GTA GTT GTG TTA CGG TAT CGA ATA TTA GAA TAT GCG GTT GTT TAA TTT GTT GGG TTA CGA GAT TAT GAA GGA GGT GTT GGA GTA GGT CGG CGT TTG GAT-3′. Within the sequence, the analyzed methylation segment was 5′-TTG TTT TTT CGG TGT TTC GTT TTT TCG CGT TTT AGT CGT CG-3′ pink-marked), the F primer was 5′-GAA AAT TTT GGT TGT GTT TTA GTA A-3′ (blue-marked), the S primer was 5′-GTT GTT AGT TTT T-3′ (blue-marked), and the R primer was 5′-GAG ATT ATG AAG GAG GTG TTG GAG T-3′ (blue-marked). Methylated sites to be analysed were pink-marked and S primers were blue marked.

*P16 primers*. Sequences were synthesized by Shanghai Sangon Biotech Co., Ltd. (Shanghai, China), as previously described (16). The wild type primers (P16-W) were 5′-CAG AGG GTG GGG CGG ACC CC-3′ and 3′-CGG GCC GCG GCC GTG G-5′, with an amplification product length (APL) of 140 bp. The methylation specific primers (P16-M) were 5′-TTA TTA GAG GGT GGG GCG GAT CGC-3′ and 3′-GAC CCC GAA CCG CGA CCG TAA-5′, with an amplification length of 150 bp. The methylation nonspecific primers (P16-U) were 5′-TTA TTA GAG GGT GGG GTG GAT TGT-3′ and 3′-CAA CCC CAA ACC ACA ACC ATA A-5′, with an APL of 151 bp.

#### Iodine-125 seed implantation

The animals were randomized into a control group and an experimental group (n=25 in each). The control group was implanted with blank seeds (intensity of radioactivity, 0 MBq) and the experimental group was implanted with iodine-125 seeds (14.8 MBq). At 20 days after implantation, the animals were sacrificed using cervical dislocation. The tumors were harvested and weighed (Wt). Spherical tumors with a diameter of ~1.5 cm (using the seed source as the center) were obtained and then immediately stored at −80°C.

#### DNA extraction

Approximately 10 *μ*g of DNA were diluted in 18 *μ*l of sterile water. Following water bath denaturation, the sample was applied to sodium bisulfate treatment liquid for 12–16 h in a water bath in the dark as well as purification. The DNA sample was eluted into 50 *μ*l of sterile water, then 11 *μ*l of NaOH was added and mixed. After 15 min in a water bath at 37°C, 166 *μ*l of 5 M ammonium acetate, 750 *μ*l of dehydrated alcohol and 200 *μ*l of isopropanol were added. The solution was subject to 2–4 h of precipitation at −20°C, centrifugation and then atmospheric drying. The obtained products were placed into 50 *μ*l of TE buffer and kept at −20°C. Assays and the determination of A260/A280 ratios were performed using a spectrophotometer (Biowave DNA; WPA Company, Taunton, UK).

#### Sulfite management

Approximately 10 *μ*l of DNA liquid was managed according to the instructions of a CpGenome™ Turbo Bisulfite Modification kit (Millipore, Shanghai, China). After water bath denaturation, at 50°C the sample was applied to sodium bisulfate treatment liquid for 12–16 h of a water bath away from light as well as purification. DNA elution buffer (30 *μ*l) was used to recycle the DNA.

#### Polymerase chain reaction (PCR)

PCR was performed using TransStart Taq DNA polymerase (TransGen Biotech Company, Beijing, China). The reaction system at 30 *μ*l contained 10 *μ*l of DNA, 2 *μ*l of the F primer, 2 *μ*l of the R primer, 4 *μ*l of 10X TransStart Taq buffer, 2 *μ*l of 1.5 mM dNTPs, 0.5 *μ*l of DNA polymerase and 9.5 *μ*l of ddH_2_O. The amplification conditions consisted of 94°C for 5 min, 35 cycles of 94°C for 30 sec, 56 °C for 30 sec and 72°C for 30 sec, and 72°C for 5 min. The amplification products were subjected to agarose gel electrophoresis.

#### Methylation detection

Approximately 20 *μ*l of the PCR products were analyzed for methylation using a Pyromark ID96 instrument and a single-stranded DNA purification PyroGold reagent kit (Biotage, Uppsala, Sweden). The procedure was conducted according to the manufacturer’s instructions.

#### Methylation indices

Approximately 20 *μ*l of DNA solution, 3 *μ*l of magnetic beads (Millipore, Shanghai, China), 40 *μ*l of binding buffer (Millipore) and 17 *μ*l deionized water were mixed for premixed liquid. The liquid was applied to a PCE plate, sealed and then agitated for 15 min to allow sufficient binding of the DNA strands and magnetic beads. A Pyromark plate solution was prepared with 2.1 *μ*l sequencing primers (10 *μ*M) and 24.5 *μ*l of Pyromark Anneling buffer. The solution was added to a PCR instrument. Three-minute annealing at 80°C was performed. Denaturation solution, dehydrated alcohol, wash buffer (Millipore) and deionized water were added to the bench board (Millipore). The two types of solution were respectively placed into their assigned positions and a detecting head was used to attract the DNA single strands in the Pyromark solution. The Pyromark plate solution was dislodged and added to the PCR instrument for 2 min for annealing at 80°C. dNTP, substrates and DNAase were correspondingly added to reagent cabins. The prepared reagent cabins and Pyromark plate solution were detected by a pyrosequencing instrument (17).

#### Statistical analysis

Data are presented as mean ± standard deviation. Statistical analyses were performed using SPSS 16.0 software (SPSS, Inc., Chicago, IL, USA). ANOVA was used to compare the SFRP_2_ methylation indices and the difference was tested using a Student’s t-test. The difference in the incidence rates of P16 methylation between groups was analyzed using a Chi-square test. P<0.05 was considered to indicate a statistically significant result.

## Results

### 

#### SFRP_2_ methylation

**Figure 1.**
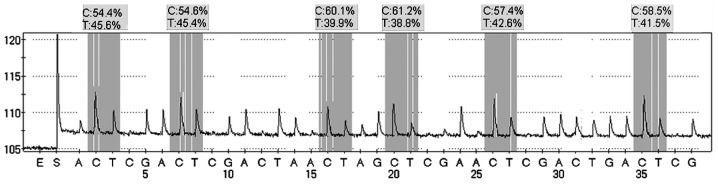
SFRP_2_ methylation in the experimental group. The gray-marked segments are the six methylation sites. The percentage after the letter C indicates the methylation level at the site. The methylation index (Mtl) is the mean methylation percentage at the six sites (the Mtl in this figure = 54.4 + 54.6 + 60.1 + 61.2 + 57.4 + 58.5%/6 = 57.7%).

According to the SFRP_2_ methylation detection results, the six C/T sites were the methylated sites. The methylation levels at these six sites were 54.4%, 54.6%, 60.1%, 61.2%, 57.4% and 58.5%, respectively. The methylation index (Mtl) (18) was 57.7%. The results are shown in Fig. 1, as follows: C1: C/TGAC/TGACTAAAAC/TGC/TGAAAAAAC/TGAAACACC/TGAAAAAACAA.

#### Methylation-specific PCR (MSP) analysis of P16

**Figure 2.**
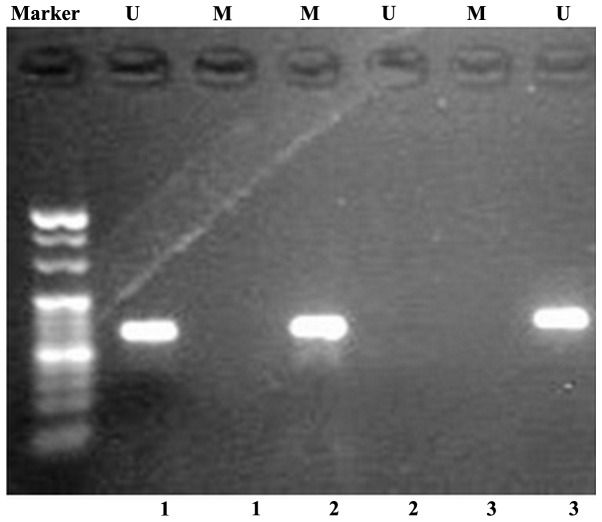
Results of P16 PCR amplification, where U refers to the primers of non-methylated specific amplification products. 1, human normal colon tissue. 2, tumor tissues in the control group. 3, tumor tissues in the experimental group. U, methylation nonspecific amplification products (151 bp). M, methylation-specific amplification products (150 bp).

DNA was amplified in the experimental and control groups following the modification of the tumor tissues using the P16-M and P16-U primers. In the experimental group, 10 samples exhibited positive P16-M amplification and 15 exhibited positive P16-U amplification. In the control group, 14 samples exhibited positive P16-M amplification and 11 exhibited negative results. The MSP amplification products were subjected to electrophoresis and the gels were photographed. The results are shown in Fig. 2.

#### Statistical analysis of P16 methylation

In the control group, methylation in the P16 promoter region was detected in 14 samples (56%). In the experimental group, P16 methylation was demonstrated in 10 samples (40%), which demonstrated a significant difference between the groups (P<0.05; Table I and Fig. 2).

## Discussion

The development of colorectal cancer involves numerous factors and multiple steps. This process is invariably accompanied by noticeable genetic changes. However, genetic changes do not provide a complete explanation for the development of all colorectal cancers. As epigenetics advances, it has been demonstrated that DNA methylation plays a critical role in the development of colorectal cancer. The joint action of DNA methylation and genetic changes leads to tumorigenesis (18). SFRP_2_ and P16 are tumor suppressor genes that participate in cell cycle regulation. Their normal expression inhibits cell division and growth (19). However, their hypermethylation may lead to tumorigenesis.

Iodine-125 seeds have been applied in the treatment of prostate carcinoma, pancreatic cancer, brain cancer and colorectal cancer in clinical practice. Iodine-125 seeds release γ-rays with an energy of ~35.5 keV and an effective irradiation radius of ~1.7 cm and, therefore, provide a low-dose irradiation treatment. The main effect of iodine-125 seeds on tumors is damage to the DNA duplex structure by irradiation, resulting in apoptosis (20).

This study aimed to investigate the effects of iodine-125 seeds on the methylation of SFRP_2_ and P16 in colorectal cancer. Iodine-125 seeds were used as the γ-ray source to act on tumor cells and the changes in SFRP_2_ promoter methylation were quantitatively detected. The results showed that the level of SFRP_2_ methylation decreased following iodine-125 irradiation. The methylation index (Mtl) of the experimental group (0.67±0.05) demonstrated a significant difference compared with that of the control group (0.84±0.07; Student’s t-test: P<0.05). Furthermore, the CpG island methylation in the P16 promoter region was detected using MSP. The results showed that the methylation positive rate of the experimental group (40%) was significantly lower than that of the control group (56%; P<0.05). This result indicates that iodine-125 seed irradiation decreases the P16 methylation rate in tumor tissues to a certain extent to inhibit tumors. This was in accordance with our predictions: iodine-125 seeds downregulate tumor suppressor gene promoter methylation to inhibit the proliferation and growth of tumor cells. In addition, this study showed that certain tumor tissues implanted with iodine-125 seeds, and some without such an implantation, displayed the coexistence of methylated and non-methylated amplification products. This phenomenon is likely due to the coexistence of different cell subsets in tumors, the existence of normal tissues in tumors or the methylation of one allele rather than another.

The mechanisms underlying the downregulatory effect of iodine-125 seeds on the methylation level of tumor suppressor genes remain uncertain. The possible mechanisms may include the following: i) γ-rays directly damage the double strands of DNA, and ii) γ-rays downregulate the expression of DNA methyltransferases. DNA double strand disruption is the main form of DNA damage caused by γ-rays. DNA damage is accompanied by the repair startup. Although disrupted DNA may be restored by means of recombination and partial fragment resection, the already methylated cytosine may also be resected during the process, which reduces the level of methylated cytosine and reduces the level of CpG island methylation. By contrast, the generation and maintenance of methylation requires the participation of methyltransferases. Therefore, the downregulated expression of the associated enzymes caused by γ-rays results in a reduced methylation level. Iodine-125 seed irradiation at a dose of 4 Gy decreases the protein expression of DNMT1 and DNMT3b in SW-1990 human pancreatic cancer cells (13). Another study has demonstrated that reduced methylation is accompanied by the downregulation of DNMT expression (21).

At present, the majority of studies concerning the effects of γ-rays on gene methylation are *in vitro* tests in cell. However, the environment for cell growth *in vitro* is different from that *in vivo*. The accumulated irradiation energy following iodine-125 permanent implantation in clinical practice is also different from a dose administered in trials. Therefore, whether the results of these studies reflect the changes in gene methylation *in vivo* remains unclear. At present, studies concerning the effect of iodine-125 seeds on tumors primarily focus on DNA strand disruption and cell apoptosis. To the best of our knowledge, the present study is the first to investigate the regulatory effect of iodine-125 seeds on the promoter methylation of tumor suppressor genes by quantitating the changes in DNA methylation. The study provides a practical basis for the application of iodine-125 seed implantation in the treatment of tumors. However, since iodine-125 seed implantation is a new technique, its molecular biology remains to be explored.

## Figures and Tables

**Table I.** P16 methylation in the experimental and control groups.

Group	No. of mice	Methylation (%)	Non-methylation (%)
Experimental	25	10 (40)	15 (60)
Control	25	14 (56)	11 (44)
Total	50	24 (48)	26 (52)
